# A Rapid and Sensitive LC−MS/MS Method for the Quantitation of Physalin A with Special Consideration to Chemical Stability in Rat Plasma: Application to a Pharmacokinetic Study

**DOI:** 10.3390/molecules27217272

**Published:** 2022-10-26

**Authors:** Yang Li, Na Zhao, Tingting Zhang, Xinchi Feng

**Affiliations:** 1 School of Chinese Materia Medica, Tianjin University of Traditional Chinese Medicine, Tianjin 301617, China; 2 State Key Laboratory of Component-Based Chinese Medicine, Tianjin University of Traditional Chinese Medicine, Tianjin 301617, China

**Keywords:** physalin A, LC–MS/MS, stability, pharmacokinetics

## Abstract

Physalin A is a promising natural product with excellent anti-inflammatory and anti-tumor activities. However, the pharmacokinetic profile of physalin A is still unclear. In this study, a rapid and sensitive analytical method based on LC–MS/MS for the quantitation of physalin A in rat plasma with special consideration to its chemical stability was developed and validated. To avoid the degradation of physalin A, the separation of plasma was conducted at 4 °C directly after the blood samples were collected. Meanwhile, plasma samples were immediately precipitated with acetonitrile containing tolbutamide (internal standard, IS) and the pH of the supernatant was adjusted to 1.5 with formic acid. Chromatographic separation of physalin A and IS was achieved on an ACQUITY UPLC BEH-C18 column (2.1 × 50 mm, 1.7 μm) using 0.1% formic acid and acetonitrile as mobile phase delivered at 0.3 mL/min in a gradient elution mode. Physalin A and IS were detected through negative ion electrospray ionization in multiple reaction monitoring (MRM) mode. The MS/MS ion transitions for physalin A and IS were *m*/*z* 525.1–148.9 and *m*/*z* 269.8–169.9, respectively. The developed method showed good linearity over the range of 2.00–400 ng/mL. This method was successfully applied to the pharmacokinetic study of physalin A in rats following its intragastric administration and the findings were beneficial for future studies of physalin A.

## 1. Introduction

**Figure 1:**
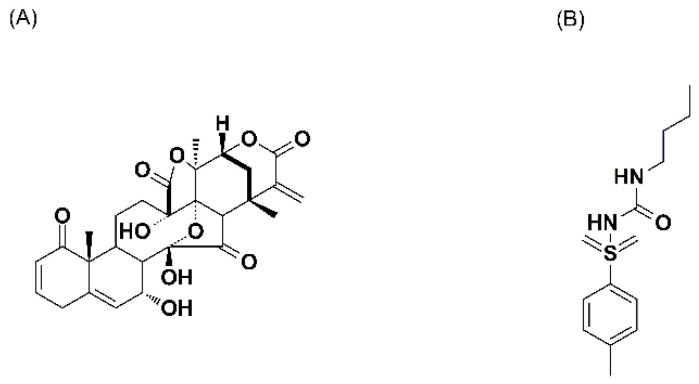
The chemical structures of (**A**) physalin A and (**B**) tolbutamide (IS).

Physalin A (Figure 1) is a promising natural product with excellent anti-inflammatory and anti-tumor activities, which is isolated from the calyces of *Physalis alkekengi* L. var. *franchetii* (Mast.) Makino, a Chinese herb named Jindenglong [1,2,3]. As the first member of physalins (a novel class of steroids), physalin A was isolated in 1969 and its 13,14-seco-16,24-cycloergostane skeleton was confirmed using X-ray crystal analysis [4,5]. In China, Jindenglong has been used for years to treat various diseases, including sore throat, cough, hepatitis, eczema, tonsillitis, pharyngitis, tumors and urinary problems [1,6,7,8,9,10,11]. Among all the physalins isolated from Jindenglong, physalin A is the most abundant one and retains the main anti-inflammatory and anti-tumor effects of Jindenglong [1,3,12,13,14,15,16,17,18]. Our previous study proved that physalin A remarkably inhibited the production of nitric oxide in lipopolysaccharide-activated macrophages [1]. Mechanism studies revealed that physalin A exhibited anti-inflammatory activity via modifying IKKβ through a Michael addition reaction [13]. Physalin A was also reported to exhibit significant growth inhibitory effects against several tumor cell lines, and A375-S2 was the found to be the most sensitive one [3,14,15,16,17,18]. It was worth mentioning that the growth of normal human peripheral blood mononuclear cells was not affected by physalin A and toxicity of physalin A on normal cells was lower than that of 5-fluorouracil or paclitaxel [15]. To sum up, physalin A could be considered a promising drug candidate, and it is necessary to develop a reliable analytical method to study the pharmacokinetic properties of physalin A for a better understanding of its pharmacological effect.

Until now, only two analytical methods have been reported for the quantitation of physalin A based on liquid chromatography–tandem mass spectrometry (LC–MS/MS) [19,20]. However, these two methods were designed for the simultaneous determination of several compounds including physalin A in rat plasma (six and five components, respectively) after administration of Jindenglong extracts, and they have some disadvantages. The method reported by Zheng et al. required a relative long chromatographic run time (8 min per sample), and 50 μL plasma was required to obtain the lower limit of quantification (LLOQ) of 4.7 ng/mL. Guo et al. developed a faster LC–MS/MS for the determination of physalin A in rat plasma with a total run time of 4 min. However, 100 μL plasma was needed to achieve the detection limit of physalin A at 2.0 ng/mL. Furthermore, physalin A contains lactone ring moieties, and some lactone drugs have been found unstable in bio-samples [21]. Additionally, reflux of physalin A in 50% methanol solution can form a C (14)-O-C (27) acetal bond and generate physalin N [22]. Our preliminary study showed that the degradation of physalin A was about 30% after incubation with buffer solution (pH 7.4) for 4 h at 37 °C. However, in the reported methods, both storage and pre-analytical handling of plasma samples were conducted with no special consideration to the possibility of physalin A degradation. Hence, the plasm concentrations of physalin A obtained from these two methods were seriously underestimated. There is an urgent need to develop an analytical method with effective strategies to stabilize physalin A in rat plasma samples.

In this work, a rapid and sensitive LC–MS/MS method for the quantitation of physalin A in rat plasma was developed and successfully used for the study of the pharmacokinetic profiles of physalin A in rats. As far as we know, this is the first study of an analytical method for the quantitative determination of physalin A with special consideration to its chemical stability in rat plasma.

## 2. Results and Discussion

### 2.1. Investigation of the Chemical Stability of Physalin A

**Figure 2:**
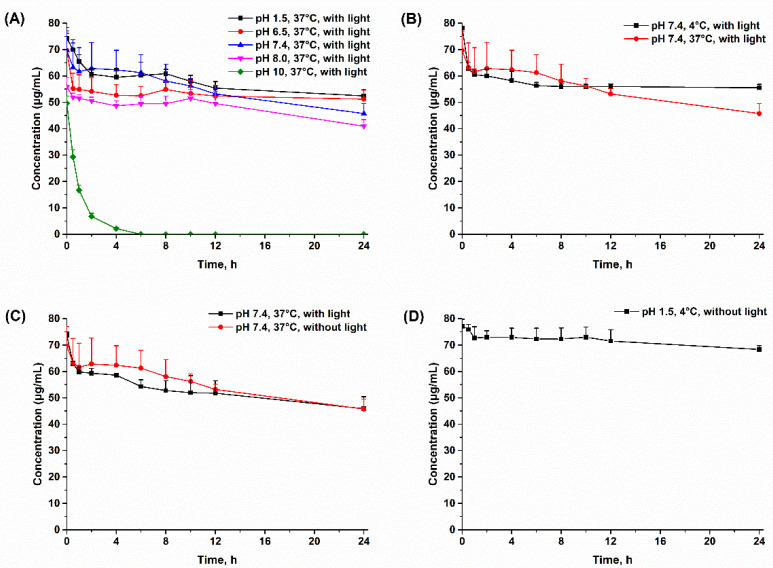
Effects of different conditions on the degradation of 80 μg/mL physalin A at 24 h. Data are presented as mean ± SD (*n* = 6). (**A**) Physalin A was incubated with light in buffer solutions with different pH values at 37 °C; (**B**) Physalin A was incubated with light in a buffer solution of pH 7.4 at 4 °C and 37 °C; (**C**) Physalin A was incubated in a buffer solution of pH 7.4 at 37 °C, with or without light; (**D**) Physalin A was incubated in a buffer solution of pH 1.5 at 4 °C, without light.

Early in 2002, researchers reported that physalin A in 50% methanol solution underwent tautomerization and generated physalin N [22]. Recently, the mechanism of physalin’s tautomerization was detailed, revealed by Zou’s group [23]. It was reported that H_2_O was the key factor leading to the instability of physalins and even a small amount of H_2_O (0.5%) in solution triggered interconversion [23]. Based on these reports, there is no doubt that physalin A is unstable in whole blood or plasma, which contains a large amount of H_2_O. Thus, in this study, it was quite necessary that plasma samples were separated immediately after the collection of blood samples and the protein precipitation procedure was conducted immediately after the plasma was obtained. After the extraction of physalin A in plasma, emphasis was placed on the stability of the extracts. In the present study, the stability of physalin A solutions (80 μg/mL) with different pH values (1.5, 6.5, 7.4, 8, 10) at 4 °C and 37 °C, with light and without light was investigated. As shown in Figure 2A, physalin A was more stable in acidic buffer (pH 1.5) and degraded faster with the increase of pH value. At a pH of 10, the concentration of physalin A rapidly degraded to 0 within 5 h. As showed in Figure 2B, the concentration of physalin A solution stored at 4 °C for 24 h was 50.52 ± 1.26 μg/mL, which was significantly greater (*p* < 0.05) than that stored at 37 °C (45.77 ± 3.77 μg/mL). Thus, 4 °C was more conducive to the stability of physalin A than 37 °C. According to Figure 2C, no significant difference (*p* > 0.05) was observed between physalin A stored with and without light at 12 or 24 h. However, the concentration of physalin A solution stored for 6 h without light (61.27 ± 6.72 μg/mL) was significant greater (*p* < 0.05) than that stored with light (54.29 ± 2.57 μg/mL), indicating that the stability of physalin A without light was slightly better. Finally, we investigated the degradation of physalin A under the above three stable conditions (4 °C, without light and pH 1.5). Figure 2D showed that under the stable conditions, the degradation of physalin A was only 5% within 4 h and less than 15% for 24 h.

Based on the above-mentioned results, the following strategies were applied: (1) all processes of sample collection, processing and sampling were conducted in a way that excluded light; (2) blood samples were promptly centrifuged and the plasma samples were immediately treated to maintain an acidic pH of 1.5; (3) after treatment, samples waiting for injection were placed at 4 °C; (4) all samples were analyzed within 4 h after sample preparation.

### 2.2. Method Development

Tandem MS spectrometric parameters in the ESI source were optimized by a direct infusion of standard solution of physalin A and internal standard (IS) using a syringe pump. A stronger and more stable MS signal for physalin A and IS was observed in negative-ion mode, which was finally selected. Various fragmentors were evaluated and the best conditions for physalin A and IS were *m*/*z* 525.1–148.9 and *m*/*z* 269.8–169.9, respectively.

According to the previous investigation, physalin A is less stable in methanol than in acetonitrile [22]. In addition, 0.1% formic acid added into the water produced a higher sensitivity and a better peak shape. Thus, acetonitrile and 0.1% formic acid were selected as mobile phase. Gradient elution was utilized to separate physalin A and IS from the matrix in the shortest run time, which was only 3.8 min. In terms of the instability of physalin A, compared with other pre-treatment methods, the protein precipitation method was chosen due to its simplicity and time-saving.

An appropriate IS is necessary to obtain high accuracy and precision of the method. In the present study, some compounds including dexamethasone, indomethacin, omeprazole and tolbutamide were tested as IS. Under the above sample preparation conditions, the ionization response of dexamethasone and indomethacin were low and if the concentration of them increased, there would be serious residues in the UPLC system. Omeprazole showed high ionization response and eluted earlier than physalin A but unfortunately rapidly broke down under acidic conditions. The chromatographic behavior and extraction efficiency of tolbutamide were similar to those of physalin A and no endogenous and cross-talk interferences were observed; thus, tolbutamide (Figure 1B) was used as the IS for this method.

### 2.3. Method Validation

#### 2.3.1. Selectivity

**Figure 3:**
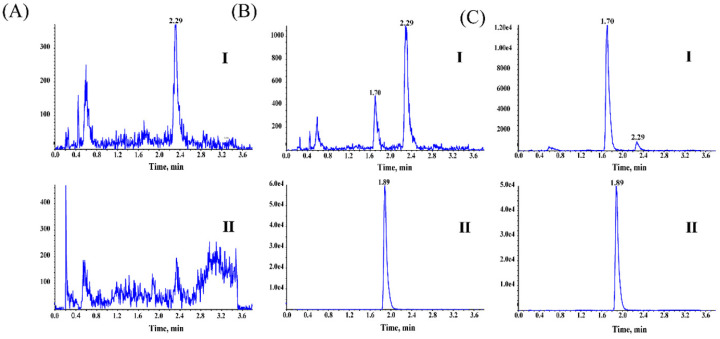
MRM chromatograms of physalin A and IS from rat plasma. (**A**) Blank rat plasma; (**B**) blank plasma samples spiked with physalin A (at LLOQ level) and IS; (**C**) real plasma samples obtained from a rat following administration of 32 mg/kg physalin A. I-Physalin A; II-IS.

The selectivity of the assay was examined by comparing the chromatograms of six different blank rat plasma samples and the corresponding plasma samples spiked with physalin A (2.00 ng/mL) and IS, and real plasma samples obtained after an oral administration of physalin A to rats. Representative chromatograms are shown in Figure 3. There was no significant endogenous substance that interfered with the determination of physalin A and IS, which were eluted at 1.70 and 1.89 min, respectively.

#### 2.3.2. Linearity of Calibration Curve and LLOQ

Calibration curves were constructed using least-squares linear regression (1/x^2^) of peak area ratios (y) of physalin A to IS versus the nominal concentration (x). Good linearity was obtained for physalin A (2.00–400 ng/mL) with correlation coefficient r ≥ 0.9982. The representative regression equation for physalin A was y = 0.00419x + 0.000833.

LLOQ was defined as the lowest concentration of physalin A that can be quantitatively determined in rat plasma with acceptable precision and accuracy (relative standard deviation (RSD%) < 20% and the relative error (RE%) within ±20%). With the present method, the LLOQ of physalin A was 2.00 ng/mL, at which the RSD was 8.7% and the RE was within ±5.8%.

#### 2.3.3. Precision and Accuracy

The accuracy and precision of the intra- and interday assays were evaluated on three validation batches, each containing six replicates quality control (QC) samples at low, medium and high concentration levels. Precision was expressed as RSD and accuracy was defined as RE. As shown in Table 1, the RSD values for intra- and interday assays were less than 8.1% and RE values were in the range of 0.9% to 5.1%. All results were within the acceptable range and the method was precise and accurate.

#### 2.3.4. Extraction Recovery and Matrix Effect

The extraction recoveries were determined at three QC levels (*n* = 6) by comparing peak areas of regular QC samples with those of the spike-post-extracted samples (*n* = 6). The spike-post-extraction samples were prepared as follows: 20 µL of acetonitrile was added to 20 µL blank plasma and the mixtures were extracted with 80 µL of acetonitrile by vortex-mixing for 1 min. After centrifugation at 14,000 rpm for 5 min at 4 °C, 60 µL supernatant was taken into another Eppendorf tube. A quantity of 20 µL of QC working solutions containing the IS (30.0 ng/mL) and 20 µL formic acid were added to the supernatant followed by vortex-mixing for 1 min. The acidified mixtures were vortex-mixed for 1 min and centrifuged at 14,000 rpm for another 5 min at 4 °C. The matrix effect was evaluated by comparing the peak areas of regular QC samples (*n* = 6) to QC samples that were treated with water instead of blank plasma.

As shown in Table 1, the extraction recoveries for physalin A were within the range of 53.9–61.8% with the RSD lower than 7.7%. The matrix effect of physalin A at three QC levels were in the acceptable range of 80–120%, whereas the RSD did not exceed 15%.

#### 2.3.5. Stability

The stability of the physalin A in rat plasma was carried out by analyzing QC samples (*n* = 6) at the low and high concentrations. Since the plasma samples were processed immediately after collection, the stability of this method was only evaluated by analyzing post-extraction QC samples stored in the automatic sampler (4 ± 5 °C) for 0, 2, 4, 8, 12 h. Stability results are summarized in Table 2. The RSD values ranged from 2.6% to 9.7% and RE values ranged from −5.2% to 5.2%. In brief, physalin A exhibited good stability in our study.

### 2.4. Pharmacokinetic Study

**Figure 4:**
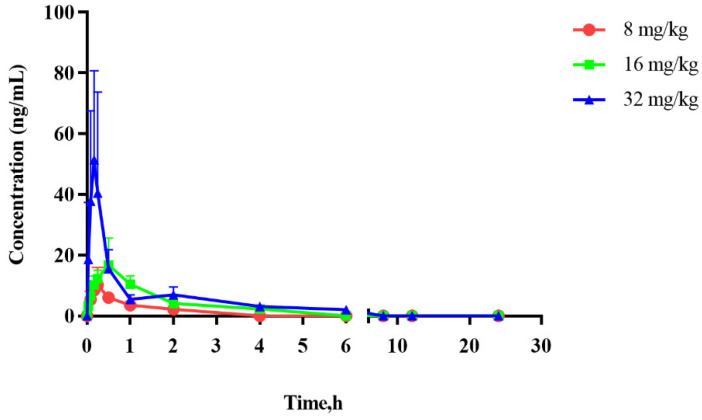
Plasma concentration–time curves of physalin A after a single dose of physalin A at 8, 16 and 32 mg/kg to rats (mean ± SD, *n* = 6).

Pharmacokinetic study of physalin A in rats after a single oral dose of physalin A at 8, 16 and 32 mg/kg was conducted using the developed and validated method. Figure 4 shows the mean plasma concentration–time profiles, and the main pharmacokinetic parameters are summarized in Table 3. After 5 min intragastric administration physalin A could be detected in plasma and reached its peak concentration (C_max_) between approximately 0.15 to 0.50 h, which indicated that physalin A was rapidly absorbed. It can be seen that physalin A was rapidly eliminated after the C_max_ was reached, with the elimination half-life (t_1/2z_) ranging from 0.67 to 1.60 h. The AUC increased with increasing doses among the three doses of treatments. Pharmacokinetics of physalin A appeared linear over the dose range of 8 to 32 mg/kg.

## 3. Materials and Methods

### 3.1. Reagents and Materials

Physalin A (purity ≥ 95%) was isolated from *Physalis alkekengi* L. var. *franchetii* (Mast.) Makino in our laboratory. The structure and purity of physalin A were affirmed based on MS, ^1^H NMR, ^13^C NMR and HPLC assays [column: Aglient ZORBAX SB-C18, 4.6 × 150 mm, 5 μm; solvent phase: methanol–H_2_O (60:40)] [1]. Tolbutamide (IS) (purity ≥ 98%) was obtained from Shanghai Yuanye Bio-Technology Co., Ltd. (Shanghai, China). Formic acid (LC/MS-grade) was obtained from Shanghai Macklin Biochemical Co., Ltd. (Shanghai, China). Acetonitrile (LC/MS-grade) was obtained from Sigma-Aldrich (St Louis, MO, USA). All other reagents or solvents were of analytical grade.

### 3.2. Investigation of the Chemical Stability of Physalin A

A Waters 2695 HPLC system was used to investigate the chemical stability of physalin A. Chromatographic separation was performed on a COSMOSIL PBr column (250 mm × 4.6 mm, 5 μm), operated at 37 °C. The mobile phase was 50% acetonitrile, and the flow rate was 1 mL/min. The detection wavelength was set at 220 nm and the injection volume was 20 μL. The method was validated in terms of selectivity, linearity (50.00 to 1000 μg/mL), accuracy and precision.

Standard stock solutions of physalin A were dissolved in acetonitrile at a concentration of 1.0 mg/mL. A series of buffer solutions (pH values of 1.5, 6.5, 7.4, 8.0, 10) were prepared with appropriate amount of HCl, KH_2_PO_4_, K_2_HPO_4_, NaHCO_3_, Na_2_CO_3_ or NaOH. Physalin A samples (800 μg/mL) for chemical stability study were prepared by diluting the stock solution of physalin A with corresponding buffer solutions. The samples were stored at different conditions (4 °C or 37 °C, with or without light) and the concentrations of physalin A in samples were determined after the samples were stored for 0, 0.5, 1, 2, 4, 6, 8, 10, 12, 24 h (*n* = 6). Statistical differences of means were determined using one-way analysis of variance (ANOVA) and were considered to be significant at a level of *p* < 0.05.

### 3.3. Animals

Male Sprague–Dawley rats (Beijing Military Medical Science Academy of the PLA, Beijing, China) weighted 200–220 g were involved in the pharmacokinetic study, approved by the Animal Ethics Committee of Tianjin University of Traditional Chinese Medicine. Rats were housed in a controlled environment (22 ± 2 °C and a 12 h light-dark cycle) with unlimited access to standard rat diet and water before the experiment, and fasted for 12 h prior to the experiment.

### 3.4. Chromatographic and Mass Spectrometric Conditions

The LC–MS/MS system utilized an ACQUITYTM UPLC (Waters) coupled to a QTRAP triple quadrupole mass spectrometer (AB SCIEX 5500). An ACQUITY UPLC BEH-C18 column (2.1 × 50 mm, 1.7 μm) equipped with an ACQUITY UPLC C18 guard column was used for chromatographic separation. The mobile phase A was 0.1% formic acid in water, and mobile phase B was acetonitrile. The flow rate of the mobile phase was set at 0.3 mL/min. The gradient elution was conducted with the following conditions: 0–2.0 min, 10–95% B; 2.0–2.8 min, 95% B; 2.8–3.0 min, 95–10% B; 3.0–3.8 min, 10% B. The injection volume was 2 μL and the temperature of the auto-sampler tray was set at 4 ± 5 °C.

Electrospray ionization (ESI) source set in negative ionization mode was used for mass spectrometric detection. Quantitation was performed using multiple reaction monitoring (MRM) and transitions for physalin A and IS were *m*/*z* 525.1–148.9 and *m*/*z* 269.8–169.9, respectively. The conditions of the mass spectrometer were as follows: Curtain Gas, 20.00 psi; Collision Gas, 8.00 psi; IonSpray Voltage, −4500 V; Temperature, 500 °C; Ion Source Gas1, 50.00 psi; Ion Source Gas2, 50.00 psi. Table 4 shows the detailed MS parameters of physalin A and IS.

### 3.5. Preparation of Standard and Quality Control Samples

Standard stock solutions of physalin A and IS were dissolved in acetonitrile at a concentration of 1.0 mg/mL. Appropriate dilutions of the stock solutions were made with acetonitrile to obtain a series of physalin A working solutions ranging from 2.00 to 400 ng/mL and an IS working solution of 30.0 ng/mL. Quality control (QC) working solutions were prepared in pools separately at low, medium and high physalin A levels (6.00, 40.0, 300 ng/mL). All the working solutions were stored without light at 4 °C.

Calibration standard samples were freshly prepared by spiking 20 μL blank rat plasma with 20 μL working solutions of physalin A and the effective concentrations in plasma samples were 2.00, 4.00, 10.0, 25.0 50.0 100, 200 and 400 ng/mL. QC samples were prepared at three levels (6.00, 40.0, 300 ng/mL) independently in the same way. After the standard samples and QC samples were prepared, sample preparation procedure was conducted immediately to avoid the degradation of physalin A in plasma.

### 3.6. Sample Preparation

After the blood samples were collected from the rat fossa orbitalis, they were centrifuged at 4 °C immediately and the obtained plasma samples were instantly treated due to their chemical instability. Centrifuge tubes of 1.5-mL wrapped in foil were used to avoid light. When standard samples or QC samples were assayed, 80 μL acetonitrile containing IS (30 ng/mL) was added into standard samples or QC samples. When the obtained real rat plasma samples were assayed, an aliquot of 20 μL rat plasma, 20 μL acetonitrile and 80 μL acetonitrile containing IS (30 ng/mL) were added. The mixtures were then vortex-mixed for 1 min and centrifuged at 14,000 rpm for 5 min at 4 °C. A supernatant layer of 80 μL was transferred to a clean wrapped tube followed by immediate addition of 20 μL formic acid to adjust the pH to 1.5. The acidified mixtures were vortex-mixed for 1 min and centrifuged at 14,000 rpm for another 5 min at 4 °C. Afterward, 2 μL solution was subjected to the LC–MS/MS system.

### 3.7. Method Validation

The method was validated following the criteria suggested by the FDA guidance for industry [24].

### 3.8. Pharmacokinetic Study

Eighteen rats were randomly divided into three groups and intragastrically administered with physalin A prepared in 0.5% CMC-Na at a single dose of 8, 16 or 32 mg/kg. Approximately 0.1 mL of blood samples were collected into an EDTA-Na_2_-containing wrapped tube at 0, 0.033, 0.083, 0.167, 0.25, 0.5, 1, 2, 4, 6, 8, 12 and 24 h post administration. Blood samples were centrifuged at 14,000 rpm for 10 min at 4 °C to obtain the plasma samples, which were treated and analyzed immediately. Non-compartmental pharmacokinetic parameters of physalin A were calculated by Drug and Statistics (DAS) software version 3.2.8 (Shanghai University of Traditional Chinese Medicine, Shanghai, China).

## 4. Conclusions

A sensitive and rapid LC–MS/MS method was developed and validated for the quantification of physalin A in rat plasma. Compared with previously reported data, the point of our work is focused on the determination of physalin A in rat plasma with special consideration to chemical stability. Strategies that could help stabilize physalin A in plasma were applied in our study, namely, plasma samples were immediately separated and processed at 4 °C without light, and acidification of the samples was conducted to maintain a pH of 1.5 before LC–MS/MS analysis. The method provided a simple protein precipitation procedure, and the total run time was only 3.8 min per sample. Only 20 μL consumption of plasma was required and an LLOQ of 2.00 ng/mL was achieved. This method was successfully applied for the pharmacokinetic study of physalin A in rats after oral administration at single doses of 8, 16 and 32 mg/kg, and these results provide valuable information for the clinical utility of physalin A.

## Figures and Tables

**Table 1:** Accuracy, precision, extraction recovery and matrix effect for LC–MS/MS analysis of physalin A in rat plasma.

	Nominal Concentration (ng/mL)	Found Concentration (ng/mL)	Intraday RSD (%)	Interday RSD (%)	Accuracy RE (%)	Matrix Effect (%)	Recovery (%)
Physalin A	6.00	6.32 ± 0.23	3.7	8.1	2.2	92.9 ± 9.43	61.8 ± 4.78
	40.0	40.6 ± 0.69	1.7	3.2	0.9	83.0 ± 3.10	53.9 ± 2.71
	300	321 ± 6.49	2.0	6.3	5.1	85.7 ± 2.30	61.4 ± 1.65
IS	30.0	-	-	-	-	99.5 ± 2.99	88.7 ± 2.33

**Table 2:** Stability of physalin A stored in auto-sampler before LC–MS/MS analysis.

Nominal Concentration	Auto-Sampler 0 h		Auto-Sampler 2 h		Auto-Sampler 4 h		Auto-Sampler 8 h		Auto-Sampler 12 h	
(ng/mL)	RSD (%)	RE (%)	RSD (%)	RE (%)	RSD (%)	RE (%)	RSD (%)	RE (%)	RSD (%)	RE (%)
6.00	2.7	−0.3	4.4	−0.3	3.9	−5.2	9.7	−3.9	8.6	−4.3
300	4.5	3.6	4.0	5.2	4.3	4.0	4.5	2.8	2.6	0.1

**Table 3:** Pharmacokinetic parameters of physalin A in rats after a single oral administration of physalin A at 8, 16 and 32 mg/kg.

Parameters	8 mg/kg	16 mg/kg	32 mg/kg
AUC_0–t_ (mg/L·h)	7.07 ± 2.85	24.53 ± 9.90	35.41 ± 10.99
AUC_0–∞_ (mg/L·h)	9.39 ± 3.39	28.76 ± 7.72	41.65 ± 13.44
C_max_ (mg/L)	10.94 ± 5.32	17.82 ± 8.19	72.08 ± 18.15
T_max_ (h)	0.19 ± 0.07	0.50 ± 0.27	0.15 ± 0.06
t_1/2z_ (h)	0.67 ± 0.33	1.15 ± 0.34	1.60 ± 1.03
Vz/F (L/kg)	841 ± 350	964 ± 358	1706 ± 884
CLz/F (L/h/kg)	939 ± 299	581 ± 111	867 ± 375
MRT_0–t_ (h)	0.54 ± 0.18	1.18 ± 0.27	1.03 ± 0.42
MRT_0–∞_ (h)	1.00 ± 0.44	1.76 ± 0.37	1.85 ± 1.04

**Table 4:** Mass scan method parameters of physalin A and IS.

Compound	Q1	Q3	DP (V)	EP (V)	CE (V)	CXP (V)
Physalin A	525.1	148.9	−30	−10	−40	−17
Tolbutamide (IS)	269.8	169.9	−100	−10	−30	−17

## Data Availability

The data presented in this study are available in the article.

## Sample Availability

Samples of the physalin A and tolbutamide are available from the authors.
